# Hybrid Desmoplastic/Follicular Ameloblastoma of the Mandible: A Case Report and Review of the Literature

**DOI:** 10.1155/2017/7031414

**Published:** 2017-05-11

**Authors:** Masayasu Iwase, Airi Fukuoka, Yoko Tanaka, Naoyuki Saida, Eriko Onaka, Sanae Bando, Gen Kondo

**Affiliations:** ^1^Department of Dentistry and Oral Surgery, Hakujikai Memorial General Hospital, Tokyo, Japan; ^2^Department of Oral and Maxillofacial Surgery, School of Dental Medicine, Tsurumi University, Kanagawa, Japan; ^3^Department of Dentistry and Oral Surgery, Jinkokai Hospital, Kanagawa, Japan

## Abstract

Desmoplastic ameloblastoma (DA) is one of the 6 histopathological subtypes of ameloblastoma. Hybrid lesions in which histopathologically conventional ameloblastoma coexists with areas of DA are rare. A 40-year-old male was referred to our hospital complaining of a swelling in the right premolar region of the mandible. A panoramic radiograph showed an area of radiolucency with a well-defined corticated border, whereas computed tomography revealed a unilocular radiolucent lesion and buccal expansion together with cortical perforation. The lesion was treated via enucleation and curettage of the marginal bone and fenestration. A histopathological examination showed a hybrid ameloblastoma with a pronounced desmoplastic pattern and follicular changes. The patient's postoperative course has been favorable up to now, and no marked changes have been observed. We presented a case of hybrid ameloblastoma and reviewed the 36 reported cases of hybrid ameloblastoma that have been reported in the English literature.

## 1. Introduction

Ameloblastomas are common benign neoplasms that frequently arise in the molar and ramus regions of the mandible. In 2005, the World Health Organization histologically classified ameloblastomas into solid/multicystic, extraosseous/peripheral, desmoplastic, and unicystic types. Solid/multicystic ameloblastomas were further divided into follicular and plexiform types. The follicular type has 4 subtypes, the spindle cell type, acanthomatous type, granular type, and basal cell type [1]. Desmoplastic ameloblastoma (DA) is relatively rare, accounting for 4–13% of all ameloblastomas [2, 3]. It has a predilection for the anterior and premolar regions of the mandible and maxilla [2–6]. On plain radiographs, DA frequently presents as a mixed radiolucent/radiopaque lesion with diffuse surrounding features, whereas the majority of other ameloblastoma variants are predominantly radiolucent [2–6]. This makes the radiological differentiation of these two lesions challenging in many cases. Histologically, DA is characterized by the presence of extensive stromal collagenization or desmoplasia containing small nests and strands of odontogenic epithelial tissue [2–6].

In recent years, the literature has described hybrid lesions composed of DA and conventional ameloblastoma, which are histopathologically characterized by areas of follicular or plexiform ameloblastoma coexisting with areas of DA [2, 7, 8].

In the present study, we describe a novel case of hybrid ameloblastoma and present a concise review in the scientific literature to summarize the clinicopathologic characteristics of hybrid ameloblastoma.

## 2. Materials and Methods

### 2.1. Literature Review

A literature review revealed 34 cases of hybrid ameloblastoma according to database of PubMed (Table 1). The present case makes up the 35th case. Gender, age, tumor site, radiographic imaging findings, and pathological type were retrieved, collated, and analyzed.

## 3. Case Report

**Figure 1:**
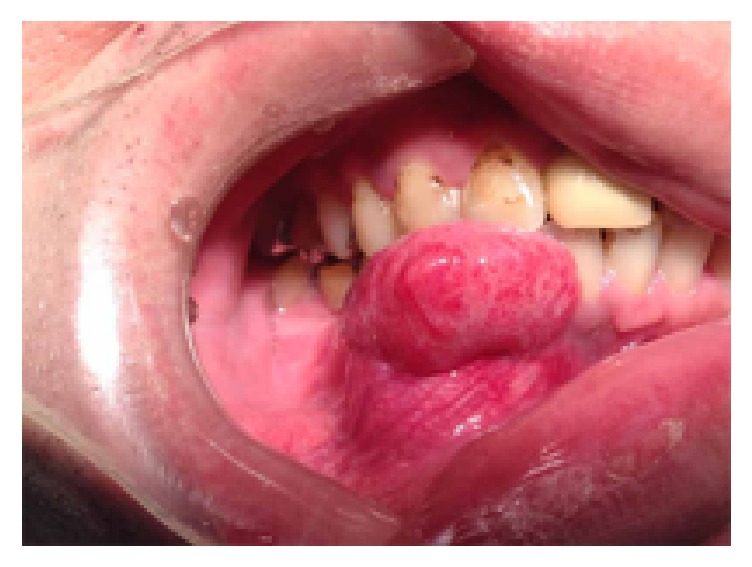
An image of the oral cavity obtained during the initial examination showing a painless mass in the lower right premolar region.

A 40-year-old male patient presented to our hospital with a complaint of swelling in the right lower premolar region (Figure 1). The patient had noticed the swelling 8 months previously, and it had slowly increased to its present size. During a clinical examination, a firm swelling of the right mandibular alveolar ridge, which extended from the lateral incisor to the second premolar, was observed (Figure 1). Patient complained of pain and tumor mass had areas of bleeding and scar formation due to accidental trauma by opposing maxillary tooth. Patient was observed to be unable to appose his lips due to the size of the mass.

**Figure 2:**
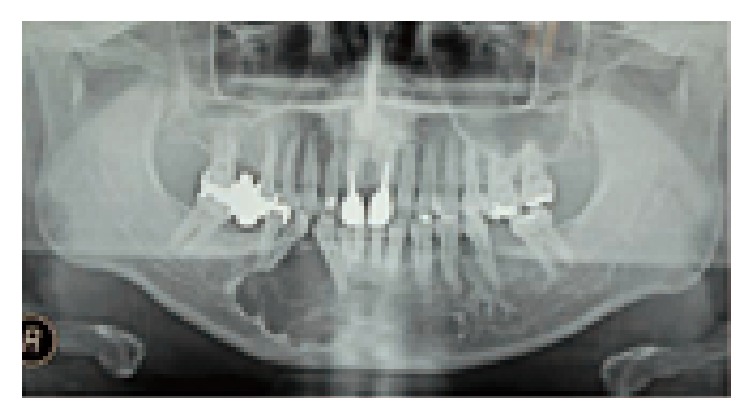
A panoramic radiograph showing a well-defined large radiolucent lesion in the right mandible.

**Figure 3:**
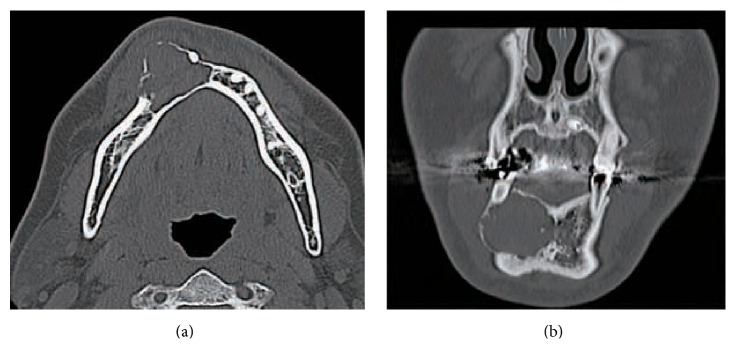
(a) Axial and (b) coronal computed tomography scans showing a large unilocular radiolucent lesion in the right mandible.

**Figure 4:**
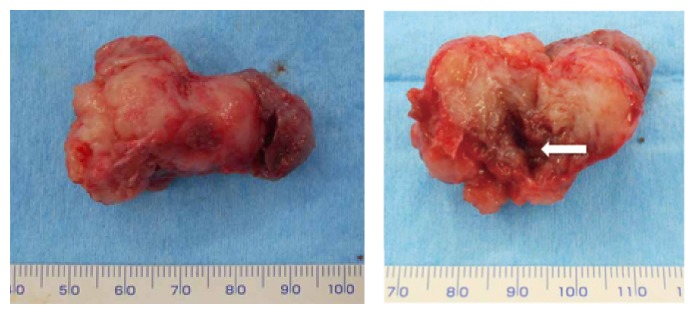
The surgical specimen had a gourd-like shape, and its cross section contained a solid portion and a cystic lesion (arrow).

**Figure 5:**
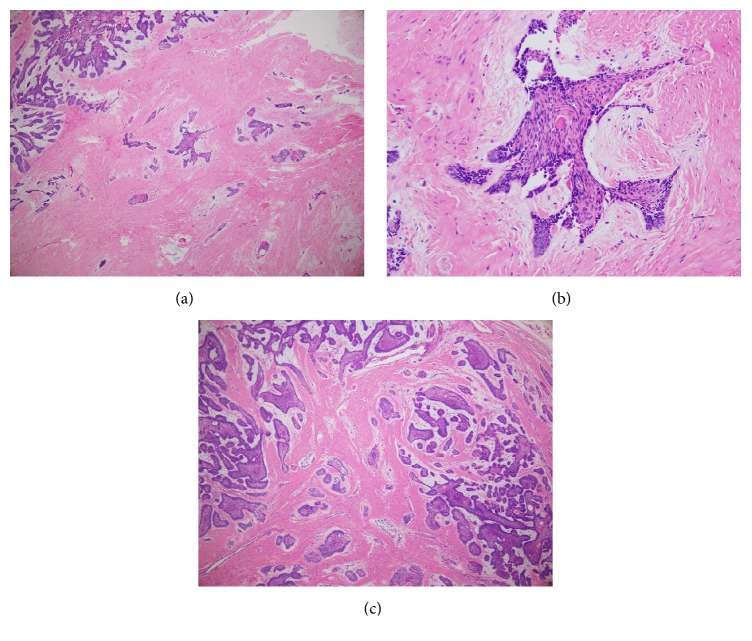
(a) A histopathological image showing a mixture of follicular and desmoplastic ameloblastoma. (×10) (b) A histopathological image showing stellate-shaped epithelial islands together with squamous metaplasia in the collagenized fibrous stroma containing connective tissue. (×40) (c) A histopathological image showing typical follicular ameloblastoma together with cystic degeneration and squamous metaplasia (×40).

**Figure 6:**
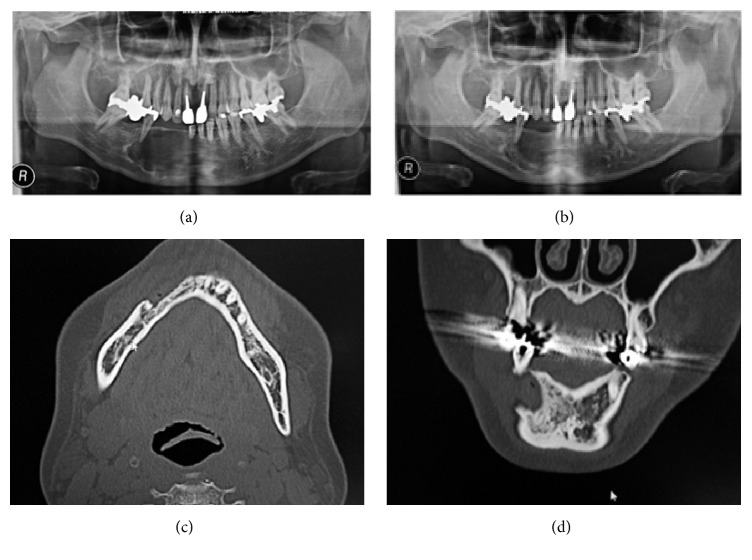
(a) A panoramic radiograph obtained just after the operation showing a well-defined large unilocular radiolucent lesion in the right mandible. (b) A panoramic radiograph and (c) axial and (d) coronal computed tomography obtained at 22 postoperative months showing that the radiopaque area had increased in size.

His past medical history was noncontributory. A panoramic radiograph revealed a radiolucent lesion with a well-defined border and root displacement of the canine and first premolar without root resorption (Figure 2). Computed tomography of the lesion showed a predominantly lytic expansile unilocular lesion measuring 40 × 35 × 35 mm (Figure 3). A clinical diagnosis of a benign tumor was made. An incisional biopsy was conducted, and the lesion was diagnosed as a benign odontogenic tumor (data not shown). Based on the histological diagnosis, extraction of the lateral incisor, canine, and first premolar was performed under general anesthesia, followed by the enucleation and curettage of the lesion without cutting the inferior alveolar nerve. The enucleated sample was firm and had an irregular shape, and its cut surface was solid, whitish, and partially cystic (Figure 4). The histopathology of the lesion was characterized by a stroma containing abundant collagen fibers and scattered tumor nests or strands composed of spindle-shaped odontogenic epithelial cells. In addition, areas of cystic degeneration and squamous metaplasia were also seen (Figure 5). The lesion histopathologically consisted of areas of desmoplastic ameloblastoma and follicular ameloblastoma and was diagnosed as a hybrid ameloblastoma. The patient's postoperative course was uneventful, and a follow-up review conducted at 22 postoperative months showed no evidence of recurrence (Figure 6).

## 4. Discussion

DA is a rare variant of ameloblastoma, with an incidence of 4% to 13% among reported ameloblastoma cases [2–6]. A painless swelling or bony expansion are the most conspicuous clinical manifestation in most cases [2–5]. The mean age of DA patients at the initial presentation ranges from 40 to 49, and DA exhibits a similar gender distribution to other ameloblastomas [2–5]. Approximately 50% of DA occur in the maxilla, with the vast majority arising in the anterior or premolar portion of the jaws, which is not consistent with the usual location of conventional ameloblastomas [2–6]. Tooth displacement which occurred in the present case is a common feature of DA. It has been reported to occur in almost 90% of cases, while root resorption has been reported in approximately a quarter of cases [3].

Histologically, the odontogenic epithelium of DA forms irregular, stellate, or follicular islands and cords, and the centers of such lesions often appear hypercellular and contain spindle-shaped or squamous cells [2]. The most striking feature which separates DA from conventional ameloblastoma is the presence of extensive stromal desmoplasia with an abundance of thick collagen fibers which compress the odontogenic epithelial islands.

The present case exhibited abundant collagen fibers in the connective tissue stroma. Marked tumor growth factor- (TGF-) *β* expression has been reported in DA but not in conventional ameloblastomas [7], implying that TGF-*β* plays a part in the formation of the desmoplastic matrix perhaps by modulating the extracellular matrix in the desmoplastic regions of DA [8]. Scientists have also indicated an association between desmoplasia and the incidence of mucous cell metaplasia in ameloblastoma [8]. Unlike with the conventional ameloblastomas, a strong positive reaction for collagen type VI has been observed in DA indicating active production of connective tissue within the stroma of DA [2]. Previous studies report that DA tumor cells display variable S-100 protein and desmin expression [9]; decreased cytokeratin 19 expression [10]; and strong p63 [11], caspase-3, and Fas [12] expression compared with conventional ameloblastoma cells. These expressions might contribute to the distinct characteristics of DA.

Hybrid ameloblastoma lesions were first described by Waldron and El-Mofty [13] as a tumor variant in which areas of follicular and plexiform ameloblastoma coexist with areas that are characteristic of DA. They speculated that secondary desmoplastic changes occur in the stroma of conventional ameloblastoma or areas of primary DA transform into a conventional ameloblastoma. The present case is a hybrid ameloblastoma composed of areas of DA and follicular ameloblastoma. The relative frequency of hybrid lesions was reported to be 4.3% in the study by Waldron and El-Mofty [13] and 3.4% and 11% by Higuchi et al. [14] and Takata et al. [7], respectively. Philipsen et al. [2] reviewed and examined the clinical characteristics of hybrid ameloblastoma lesions. Nine cases of hybrid ameloblastoma lesions in which areas of conventional ameloblastoma coexisted with areas of DA were examined with 100 cases of DA. The latter study stated that the origin of hybrid lesions is unclear and therefore it remains unknown if DA develops from conventional ameloblastoma, conventional ameloblastoma develops from DA, or hybrid ameloblastoma is a kind of collision tumor. Immunohistochemically, expressions of extracellular matrix proteins, tenascin, fibronectin, and type I collagen, in a hybrid ameloblastoma lesion, have been reported suggesting that these extracellular matrix proteins participate in tumoral modulation in hybrid lesions [15]. A previous study suggests that desmoplasia within the stromal connective tissue in DA represents a change that occurs during the maturation of conventional ameloblastomas suggesting that the follicular component arises on a desmoplastic background in hybrid ameloblastoma lesions [16]. This implies that the majority of conventional ameloblastomas are actually desmoplastic variants. Yamazaki et al. [17] have documented hybrid odontogenic tumors, including ameloblastomas and adenomatoid odontogenic tumors.

The present case comprises both components intermingled with each other, indicating that the tumor was a true hybrid lesion rather than a collision lesion. Hybrid odontogenic tumors might result from the diverse differentiation potentials of odontogenic epithelial cells, whose multipotentiality is demonstrated by the number of different histopathological varieties of odontogenic tumors. The present case had an admixture of DA component with conventional ameloblastoma tissue, which is why we consider that it was a modulated tumor rather than a collision tumor.

We performed a clinicopathological analysis of the approximately 30 cases of hybrid ameloblastoma reviewed by Rai et al. [18] and Chaitanya et al. [19] and reviewed additional cases, including the present case [20–23]. To the best of our knowledge, 35 cases of hybrid lesions composed of DA and conventional ameloblastoma have been described in the English literature, and the features of these cases are summarized in Table 1.

Several reports have described treatment algorithms for ameloblastoma [24, 25]. Many surgeons prefer a radical surgical approach in the form of resection as management of all types of ameloblastoma as it is a formidable tumor due to its local aggressive nature and its tendency to recur [24, 25]. It has been reported that DA exhibits more aggressive behavior than other types of ameloblastoma. Its aggressive nature might be due to its potential to grow to a large size; the fact that it commonly arises in the maxilla, leading to the early invasion of adjacent structures; its diffuse radiographic appearance; and the frequent histological detection of bone invasion [26]. DA displays a significantly higher recurrence rate after enucleation than after resection [3]. However, Escande et al. [27] suggested that unilocular ameloblastomas of <5 cm in diameter that contain several medium-sized “soap bubbles” should be treated within a conservative way in the form of enucleation and curettage. A previous study reports that DA tends to be less biologically aggressive than conventional ameloblastoma based on their estimated mean growth rates [5]. Darshani Gunawardhana et al. [28] also stated that DA recurs less frequently than conventional ameloblastoma. The present case was treated conservatively with the aim of maintaining quality of life by focusing on aesthetics and function. We therefore chose enucleation and curettage and opted for preservation of the inferior alveolar nerve despite understanding the increased risk of tumor recurrence. A panoramic radiograph and computed tomography showed visible new bone formation at two years after surgery and revealed no evidence of recurrence. Since a longer follow-up period of 10 years is the gold standard for ameloblastoma [24], we have planned a meticulous follow-up for this patient.

## Figures and Tables

**Table 1:** Summary of data on 35 compiled cases of hybrid ameloblastoma.

	(%)
Patient age (yr)	
Mean 44.9	
Range 18–82	
<19	3
20–29	12
30–39	24
40–49	18
50–59	28
60–69	9
>70	6
Gender	
Female	61
Male	39
Tumor site	
Maxilla	17
Mandible	83
Radiographic features	
Radiolucency	50
Radiopacity	10
Mixed radiolucency and radiopacity	40
Histopathological features	
Desmoplastic and follicular ameloblastoma	93
Desmoplastic and plexiform ameloblastoma	7
